# Increased diagnostic yield by reanalysis of data from a hearing loss gene panel

**DOI:** 10.1186/s12920-019-0531-6

**Published:** 2019-05-28

**Authors:** Yu Sun, Jiale Xiang, Yidong Liu, Sen Chen, Jintao Yu, Jiguang Peng, Zijing Liu, Lisha Chen, Jun Sun, Yun Yang, Yaping Yang, Yulin Zhou, Zhiyu Peng

**Affiliations:** 1 Department of Otorhinolaryngology, Union Hospital of Tongji Medical College, Huazhong University of Science and Technology, Wuhan, 430022 China; 2 BGI Genomics, BGI-Shenzhen, Shenzhen, 518083 China; 3 Tianjin Medical Laboratory, BGI-Tianjin, BGI-Shenzhen, Tianjin, 300308 China; 4 Department of Molecular and Human Genetics, Baylor College of Medicine, Houston, TX USA; 5 AiLife Diagnostics, 1920 Country Place Pkwy, Pearland, TX 77584 USA; 6 United Diagnostic and Research Center for Clinical Genetics, School of Public Health of Xiamen University, Xiamen, Fujian 361003 China; 7 Xiamen Maternal and Child Health Hospital, Xiamen, Fujian 361003 China

**Keywords:** Reanalysis, Variant interpretation, Multigene panel, Hearing loss, Diagnostic yield

## Abstract

**Background:**

Congenital hearing loss affects approximately 1–2 infants out of every 1000, with 50% of the cases resulting from genetic factors. Targeted gene panels have been widely used for genetic diagnosis of hearing loss. This study aims to reveal new diagnoses via reanalyzing historical data of a multigene panel, and exam the reasons for new diagnoses.

**Methods:**

A total of 210 samples were enlisted, including clinical reports and sequencing data of patients with congenital/prelingual hearing loss who were referred to clinical genetic testing from October 2014 to June 2017. All variants listed on the original clinical reports were reinterpreted according to the standards and guidelines recommended by the American College of Medical Genetics and Genomics and the Association for Molecular Pathology (ACMG/AMP). Expanded analysis of raw data were performed in undiagnosed cases.

**Results:**

Re-analysis resulted in nine new diagnoses, improving the overall diagnostic rate from 39 to 43%. New diagnoses were attributed to newly published clinical evidence in the literature, adoption of new interpretation guidelines and expanded analysis range.

**Conclusion:**

This work demonstrates benefits of reanalysis of targeted gene panel data, indicating that periodical reanalysis should be performed in clinical practice.

**Electronic supplementary material:**

The online version of this article (10.1186/s12920-019-0531-6) contains supplementary material, which is available to authorized users.

## Background

Congenital hearing loss affects approximately 1–2 infants out of every 1000, with 50% of the cases resulting from genetic factors [1]. Molecular diagnosis of hearing loss can help direct the genetic counseling and clinical management of probands and their family members [2]. One of the molecular testing methods used to diagnose hearing loss is Sanger sequencing. However, Sanger sequencing diagnoses usually begin by testing a limited number of selected genes (generally starting with *GJB2* in hearing loss), resulting in a low detection rate [3]; well-established genetic knowledge about the target population may therefore be necessary to determine which genes should be tested first. Furthermore, the genetic heterogeneity of hereditary hearing loss [4] makes sequential gene-by-gene testing unrealistic and costly.

With the advance of next generation sequencing (NGS) techniques, targeted genomic capture and massively parallel sequencing has become an important diagnostic tool for hereditary hearing loss [5]. This method can be used to examine over one hundred known deafness-related genes simultaneously. The diagnostic yield of comprehensive NGS hearing loss testing panels is close to 40% [4, 6], much higher than that of Sanger sequencing.

It was recently reported that reanalysis of genome-wide NGS data with updated knowledge can improve the diagnostic rate [7, 8]. For instance, diagnostic yield increased by 13% following reanalysis of genome-wide data from patients with severe developmental disorders [9] and by 11% in a cohort of 37 families with suspected Mendelian disorders (primarily intellectual disabilities) [10]. Reanalysis of data from gene panels targeting known disease genes will not yield new diagnoses resulting from novel gene discovery since the panels have a fixed design, but it may still be beneficial due to improvements in variant interpretation. However, reports of such reanalysis are currently lacking.

In this study, we recruited patients with congenital/prelingual hearing loss. We sought to reveal new diagnoses via reanalyzing their targeted gene panel data, and exam the reasons for new diagnoses.

## Methods

### Patients

A total of 210 patients with congenital or prelingual hearing loss, defined as detection before three years of age, were retrospectively studied. They had been referred to clinical genetic testing from October 2014 to June 2017 and consented to anonymous use of their data for scientific research. Healthy relatives of the patients were not included. The Institutional Review Board of the BGI approved this study.

### HearingCare NGS testing

After a clinical diagnosis of hearing loss, peripheral blood samples were submitted for testing with commercial exome sequencing panels, either HearingCare_127 or HearingCare_81. The two tests use the same gene panel but involve analysis of a different number of genes, with 127 genes analyzed in HearingCare_127 and 81 in HearingCare_81. The gene list is presented in Additional file 1: Table S1. All tests were performed using target capture (Agilent, Santa Clara, CA, USA) followed by sequencing on a Hiseq-2500 (Illumina, San Diego, CA, USA). The coding regions and splice sites (±10 bp) of the target genes were analyzed. Bioinformatics pipelines included alignment of sequencing reads using the Burrows-Wheeler Aligner (0.7.12) [11] and variant calling using the Genome Analysis Tool Kit (GATK 3.4) [12].

### Variant filtering and prioritization

Variant filtering and prioritization were first based on population databases (the Exome Aggregation Consortium (ExAC), the Genome Aggregation Database (gnomAD), Exome Sequencing Project v. 6500 (ESP6500), 1000 Genomes and local databases). Variants at a minor allele frequency of > 1% in either one of the databases were excluded except for hotspot variants, such as NM_004004.5(*GJB2*):c.109G > A. Then, the functional consequences of the remaining variants were predicted by Condel [13]. If no diagnosis was found for a patient through single nucleotide variants (SNVs), then copy number variants (CNV) were characterized. An in-house spreadsheet that computes the inter-sample normalized depth of coverage per exon was used starting in 2015. All reported SNVs were confirmed via Sanger sequencing and CNVs were confirmed via qPCR.

### Variant interpretation and reporting

Variants were interpreted according to the standards and guidelines published in the literature [14–16]. Each variant was classified into one of five categories: pathogenic (P), likely pathogenic (LP), variant of uncertain significance (VUS), likely benign (LB) or benign (B).

In clinical reports, P/LP variants were all listed. However, VUS in undiagnosed patients were only reported if certain conditions were met. If a VUS was found concurrently in a gene with a P/LP variant, the VUS was reported. In cases where no P/LP variant was curated, a VUS was reported based on patients’ phenotypes.

### Reanalysis workflow

**Fig. 1:**
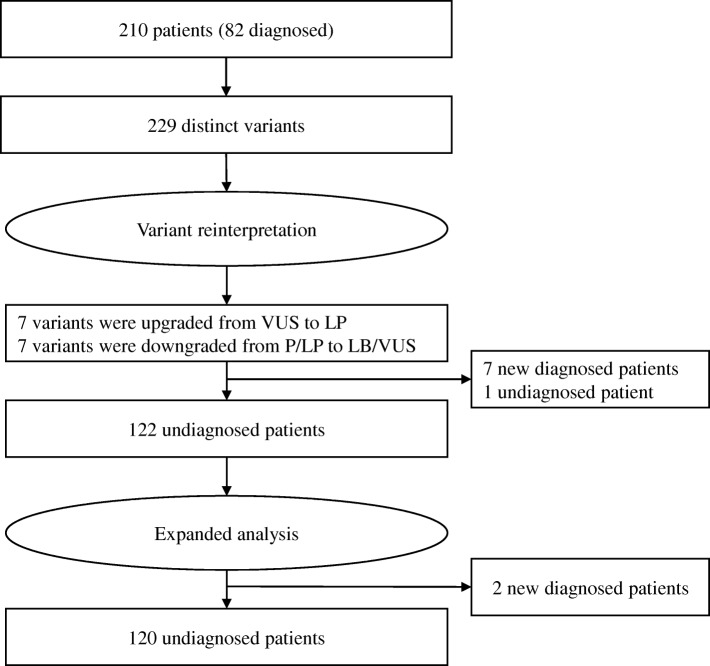
Flowchart of variant reinterpretation and expanded analysis. P, pathogenic; LP, likely pathogenic; VUS, variant of uncertain significance; LB, likely benign. The one undiagnosed patient was attributed to the downgraded of a X-linked dominant variant (NM_000495.4(*COL4A5*):c.2858G > T) from likely pathogenic to likely benign

The reanalysis workflow consisted of reinterpretation and expanded analysis (Fig. 1). The purpose of variant reinterpretation was to reassess the pathogenicity of variants (Table 1). All variants were reinterpreted according to the standards and guidelines recommended by the American College of Medical Genetics and Genomics and the Association for Molecular Pathology (ACMG/AMP) [17]. Undiagnosed cases were then processed through expanded analysis, which focused on CNV detection and examined 127 genes irrespective of the initially tested panel (Table 1). Those remained undiagnosed should be subject to periodic reanalysis.

**Table 1:** Purpose and potential sources of improvement by variant reinterpretation and expanded analysis

Step	Purpose	Potential sources of improvement
Variant reinterpretation	To reassess the pathogenicity of variants	New evidence from publications to upgrade the pathogenicity New standards and guidelines for variant interpretation
Expanded analysis	To detect copy number variants To expand analysis to other hearing loss related genes	Missed copy number variants Phenotypic heterogeneity Incorrect targeted panel tested

### Statistical analysis

For categorical data, summary data were reported as frequencies and percentages, and chi-square tests were used for between-group comparisons. A *P* value of less than 0.05 was considered statistically significant. Statistical analysis was performed with IBM SPSS Statistics, version 24 (SPSS).

## Results

### Cohort and overall results

Out of 210 patients, 49% were male. A total of 52 patients (25%) have a self-reported family history of hearing loss. The majority of cases had been tested with HearingCare_127 (83%; 174), while the remainder used HearingCare_81 (17%; 36) (Table 2).

**Table 2:** Characteristics and diagnosed yield of the study cohort

	Patients, No. (%)	Diagnosed yield before reanalysis, No. (%)	Diagnosed yield after reanalysis, No. (%)
All	210 (100)	82 (39)	90 (43)
Sex			
Male	102 (49)	42 (41)	44 (43)
Female	108 (51)	40 (37)	46 (43)
Family history			
Yes	52 (25)	21 (40)	25 (48)
No	158 (75)	61 (39)	65 (41)
Selection			
HearingCare_127	174 (83)	72 (41)	77 (44)
HearingCare_81	36 (17)	10 (28)	13 (36)
Year			
2014	10 (5)	2 (20)	4 (40)
2015	62 (30)	30 (48)	32 (52)
2016	93 (44)	33 (35)	35 (38)
2017	45 (21)	17 (38)	19 (42)

Molecular diagnoses were initially made in 82 out of 210 patients (39%). The diagnostic yields of HearingCare_127 and HearingCare_81 were 41 and 28%, respectively (*p* = 0.128). The diagnostic rate was not significantly different in patients with or without a family history (40% vs. 39%, *p* = 0.820) or in patients from different calendar years (*p* = 0.220). In diagnosed patients, *GJB2* and *SLC26A4* were the most significant contributors, present in 45 and 35% of cases, respectively. As expected, autosomal recessive inheritance was the most common inheritance pattern in congenital/prelingual hearing loss patients (Additional file 2: Table S2).

### Improvement of diagnosis

The overall diagnostic rate improved from 39 to 43% (Table 2). Of the nine new diagnoses, five patients (Patients 1–5) were upgraded in light of new evidence from studies published after the reports were first released. Pathogenic moderate evidence (PM5: a different missense change determined to be pathogenic has been seen before) from ACMG/AMP guidelines [17] was applied twice to upgrade two reports (Patient 6 and 7). In addition, a pathogenic variant with autosomal dominant inheritance was discovered in a gene which was beyond the initial analysis range (Patient 8). A CNV was found in patient 9 via expanded analysis, paired as a compound heterozygote with a single-nucleotide variant (Table 3). All the new diagnoses were attributed to the recategorization of a single variant; none were due to the recategorization of two different AR variants in the same gene. The new diagnoses were not enriched in a specific year.

**Table 3:** New diagnoses in this cohort

Patient ID	Gene	Reference	Allele 1*	Allele 2	Inheritance	Time to diagnosis	Family history	Date of report	Reason to upgrade	Reference; Published date
1	*MYO15A*	NM_016239.3	c.10245_10247delCTC; p.Ser3417del	c.9314_9315insC; p.His3106Profs*2	AR	3 y	No	September 2016	New publication	PMID29482514; February 2018
2	*MYO15A*	NM_016239.3	c.10245_10247delCTC; p.Ser3417del	c.10245_10247delCTC; p.Ser3417del	AR	27 y	YES	March 2017	New publication	PMID29482514; February 2018
3	*MYO7A*	NM_000260.3	c.3671C > A; p. Ala1224Asp	c.397dupC; p. His133Profs*7	AR	4 y	YES	July 2015	New publication	PMID26968074; April 2016
4	*MITF*	NM_198159.2	c.1021C > G; p.Arg341Gly	N/A	AD	29 y	No	August 2016	New publication	PMID29484430; January 2018 and PMID30394532; November 2018
5	*MITF*	NM_198159.2	c.1021C > T; p.Arg341Cys	N/A	AD	5 y	No	December 2014	New publication	PMID27057829; April 2016
6	*TMC1*	NM_138691.2	c.1250G > A; p. Gly417Glu	c.1250G > A; p.Gly417Glu	AR	48 y	YES	November 2015	ACMG/AMP Guidelines (PM5)	PMID25741868; May 2015
7	*CDH23*	NM_022124.5	c.1037C > G; p. Pro346Arg	c.489delA; p. Gly165Alafs*25	AR	21 y	YES	July 2015	ACMG/AMP Guidelines (PM5)	PMID25741868; May 2015
8	*PAX3*	NM_001127366.2	c.870_871insC; p. Gly292Argfs*118	N/A	AD	26 y	No	January 2017	Expanded analysis	N/A
9	*TMC1*	NM_138691.2	EX6_EX10, DEL	c.1333C > T	AR	8 y	No	December 2014	Expanded analysis	N/A

*Variants of allele 1 were those upgraded from variants of uncertain significance to likely pathogenic. PM5 denotes a pathogenic moderate criterion from the variant interpretation guidelines recommended by the American College of Medical Genetics and Genomics and the Association for Molecular Pathology. *AR* autosomal recessive. *AD* autosomal dominant. *N/A* Not applicable

In addition, one initial diagnosed case was reclassified to undiagnosed due to downgrade of a X-linked dominant variant (NM_000495.4(*COL4A5*):c.2858G > T) from likely pathogenic to likely benign (Fig. 1). The variant was interpreted and reported in March 2015. The availability of public genomic databases, such as ExAC and gnomAD [18], provided population evidence to reclassify this variant to likely benign. Moreover, we noted that the 7 upgraded variants resulted in 7 new diagnoses (7/7; 100%), whereas 7 downgraded variants only resulted in 1 patient from “diagnosed” to “undiagnosed” (1/7; 14%), reaching a significant difference (*P* < 0.001).

#### New publications (patients 1–5)

Variants that were reclassified from VUS to likely pathogenic in patients 1–5. The upgrade of NM_016239.3(*MYO15A*):c.10245_10247delCTC resulted in two probands getting diagnosed (patient 1 and 2). This variant was first reported in 2015 as a compound heterozygote with *MYO15A* c.8198A > C [19]. Because the pathogenicity of the latter variant was undetermined, the pathogenicity of c.10245_10247delCTC could not be upgraded. This changed in February 2018 when a new publication provided solid evidence that the variant segregated with nonsyndromic hearing loss in a Korean family [20]. The pathogenicity of the variant in patient 3 was also supported by evidence of segregation [21].

In patients 4 and 5, two missense amino acid changes occurred in *MITF* at the same position. The pathogenicity of p.Arg341Cys was reclassified because it was proven to be de novo and to cause prelingual hearing loss in a five-year-old girl in April 2016 [22]. The reclassification of p.Arg341Cys in turn supported the pathogenicity of Arg341Gly. Two years later, p.Arg341Gly was reported as a pathogenic variant in a Chinese family in June 2018 [23], and in a Indian family in November 2018 [24], further supporting its pathogenicity. It is worth noting that NM_198159.2(*MITF*):c.1021C > T (p.Arg341Cys) was discovered from original file of an unreported VUS in patient 5, who presented only congenital profound hearing loss when they were referred for genetic testing.

#### ACMG/AMP guidelines (patients 6–7)

Two patients were reclassified due to the application of standards and guidelines for the interpretation of sequence variants recommend by ACMG/AMP [17]. Specifically, *TMC1* p.Gly417Glu and *CDH23* p.Pro346Arg were detected in patient 6 and patient 7, respectively. Pathogenic missense changes at the positions of these two variants have been established before [25, 26], providing moderate evidence to reclassify the variants.

#### Expanded analysis (patients 8–9)

Patient 8 was clinically diagnosed with nonsyndromic hearing loss, and their sample was tested using HearingCare_81 panel. The number of genes analyzed was increased to 127 during reanalysis. An autosomal dominant frameshift variant, NM_001127366.2(*PAX3*):c.870_871insC, was discovered and is known to be the genetic cause of Waardenburg Syndrome [27]. Penetrance of individuals with Waardenburg Syndrome varied, and sensorineural hearing loss was a presenting feature in 47%~ 58% patients [27].

Patient 9 was reported to have congenital hearing loss without a family history, and his report listed only the pathogenic variant c.1333C > T in *TMC1* alone at December 2014. The expanded analysis uncovered a CNV in conjunction with this SNV. The exon-level deletion from exon 6 to exon 10 was missed in the earlier analysis because CNV analysis was not available at that time.

## Discussion

In this study, we increased the diagnostic yield of congenital/prelingual hearing loss patients from 39 to 43% by reanalysis of targeted gene panel data. Considering that the contribution of genetic factors to congenital hearing loss is around 50% [1], the improvement to 43% is significant. The residual cases might be attributable to untargeted, novel, or unknown deafness-related genes, non-exonic sequence variants, or structural variations that could be detected by whole genome/exome sequencing [9, 28, 29].

Reanalysis of targeted gene panel data for a specific disease is reliable and valuable. To date, all published reanalysis research has focused on WES data for a wide spectrum of disorders [7, 9, 10]. The diagnostic yield increased by 11~13%, mainly due to the discovery of new genes linked with the disorders [9, 10]. By comparison, in our reanalysis new evidence from publications to reclassify the pathogenicity of variants was the leading contributor of novel diagnoses. Furthermore, the dramatic increase in the number of pathogenic variants curated (9210 pathogenic variants per year) [7] reinforces the value of variant reinterpretation for such data.

Although a recent study revealed that reinterpretation of genomic test results should be performed at least every two years [30], and it is clear that periodic reanalysis of undiagnosed cases is beneficial because of the growth in knowledge linking variants and diseases, frequent reassessment is both expensive and time-consuming, rendering this approach to be static in practice. Framework to improve the efficiency of reanalysis is further required.

Consistent with previous publications, *GJB2* and *SLC26A4* were the major genetic causes of hearing loss in the Chinese population; the main contributing variants were *GJB2* c.235delC, *GJB2* c.109G > A, and *SLC26A4* c.919-2A > G [31, 32]. In our reanalysis, we upgraded a three-base deletion, NM_016239.3(*MYO15A*):c.10245_10247delCTC, from VUS to pathogenic in light of evidence that it cosegregates with the disease [20]. This variant was detected in two probands (compound heterozygous in patient 1 and homozygous in patient 2). The allele frequency was 0.299% in our in-house hearing loss patient database, whereas it was 0.06% in Eastern Asian and was not detected in other ethnic groups in the Genome Aggregation Database [18]. Together, these findings support the notion that this pathogenic allele is enriched in the Asian population.

Many challenges remain for variant interpretation in practice. First, transcript discrepancy can lead to inaccurate variant interpretation [33]. For example, an autosomal dominant variant was curated as NM_198159.2(*MITF*):c.1021C > G in patient 6, whereas it was reported as NM_000248.3(*MITF*):c.718C > G [23], creating a significant barrier to discovery for geneticists. Many genes produce multiple transcripts, and determining which should be used as a reference for evaluating the impact of a variant often presents a challenge [34]. Recently, clinically relevant transcripts of deafness-related genes were systematically curated [35], offering a path to unify the use of transcripts in analyzing hereditary hearing loss. The framework for transcript curation and selection might offer a good example of consistency in variant interpretation.

Second, genetic heterogeneity (in terms of alleles and loci) is notable in inherited diseases, especially hearing loss, making it harder to identify causal variants. Allelic heterogeneity sometimes leads to heterogeneity in the clinical phenotype. In our study cohort, patient 8 presented with congenital nonsyndromic hearing loss, leading to the use of HearingCare_81 panel, which mainly targets nonsyndromic hearing loss genes. As a result, the Chinese hotspot variant NM_004004.5(*GJB2*):c.109G > A was detected and reported in a heterozygous state. Our expanded analysis revealed a frameshift autosomal dominant variant, NM_001127366.2(*PAX3*):c.870_871insC, in the original unfiltered files. Since *PAX3* is linked with Waardenburg syndrome with variable clinical features [27], it was not included in the HearingCare_81 panel and thus not considered as the molecular etiology of this case. The heterogeneity and penetrance of the phenotype misled audiologists, resulting in this patient initially being undiagnosed.

High levels of locus heterogeneity present another challenge. In the database of Online Mendelian Inheritance in Man, over 100 genes are associated with hearing loss. This means that numerous VUS from different genes were curated and interpreted, presenting a challenge for geneticists in determining which VUS to prioritize and report when no pathogenic variant is detected. To date, professional societies have not provided specific recommendations about VUS reporting [36, 37], and reporting practices for VUS vary dramatically between different laboratories [38].

Third, the interpretation of the criteria in the ACMG/AMP guidelines is not always consistent between laboratories. For example, the application of variant frequency in publicly available population databases is recommended, whereas the cutoffs are not indicated except for benign variants alone (allele frequency > 5%) [17]. Similar ambiguity can be seen in PM3 (For recessive disorders, detected in trans with a pathogenic variant) and PP1 (Cosegregation with disease in multiple affected family members in a gene definitively known to cause the disease), in which stronger conclusions can be drawn on the basis of more data [17]. Recently, expert specifications of the ACMG/AMP variant interpretation guidelines for genes and disorders have been published [39, 40], and the guidelines are expected to continue to become more specific in the future.

Although we did not recontact the patients for the updated results yet, we noted that a number of genetic centers are recontacting patients occasionally or periodically for modified results [41], which may pose ethical and legal issues. For example, reinterpretation may upgrade a participant’s results from negative to positive. However, the participant may not want positive genomic results in their medical records for their own reasons. In this circumstance, the participant’s autonomy should be respected [42]. Recently, the American Society of Human Genetics developed a position statement to provide necessary guidance, which will facilitate researchers to appropriately operationalize patient recontact after reinterpretation of genetic and genomic research results [43].

## Conclusions

In conclusion, this work demonstrates benefits of reanalysis of targeted gene panel data from congenital/prelingual hearing loss patients. A total of 9 previously undiagnosed case obtained diagnosis, improving the overall diagnostic rate from 39 to 43%. New diagnoses are attributed to newly published clinical evidence in the literature, adoption of new interpretation guidelines and expanded analysis range. In spite of the fixed design of targeted gene panels, reanalysis of such data is still beneficial due to the improvements in variant interpretation. We propose that periodical reanalysis should be performed in clinical practice.

## Additional files

Additional file 1: **Table S1.** Gene list of diagnostic hearing loss panel (HearingCare). (DOCX 42 kb)

Additional file 2: **Table S2.** Molecular characteristics of diagnosed patients. (DOCX 26 kb)

## Abbreviations

ACMG/AMP: American College of Medical Genetics and Genomics and the Association for Molecular Pathology
AD: Autosomal dominant
AR: Autosomal recessive
CNV: Copy number variation
NGS: Next-generation sequencing
VUS: variants of uncertain significance
