# Supplementary material for: Increased diagnostic yield by reanalysis of data from a hearing loss gene panel
Source: BMC Med Genomics. 2019 May 28;12:76. doi: 10.1186/s12920-019-0531-6 (PMC6540452; doi:10.1186/s12920-019-0531-6)
Supplement: Supplementary file 1 — Table S1. Gene list of diagnostic hearing loss panel (HearingCare). (DOCX 42 kb) [file 12920_2019_531_MOESM1_ESM.docx]

**Table S1 Gene list of diagnostic hearing loss panel (HearingCare)**

| Gene | OMIM | Inheritance | HearingCare_127 | HearingCare_81 | Gene | OMIM | Inheritance | HearingCare_127 | HearingCare_81 |
| --- | --- | --- | --- | --- | --- | --- | --- | --- | --- |
| *ACTG1* | 102560 | AD | √ | √ | *MYH9* | 160775 | AD | √ | √ |
| *ATP2B2* | 108733 | AR | √ | √ | *FGF3* | 164950 | AR | √ | - |
| *CACNA1D* | 114206 | AD;AR | √ | √ | *GLI3* | 165240 | AD;AR | √ | - |
| *COL4A3* | 120070 | AD;AR | √ | - | *PAX2* | 167409 | AD | √ | - |
| *COL4A4* | 120131 | AR | √ | - | *PRRX1* | 167420 | AD;AR | √ | - |
| *COL2A1* | 120140 | AD;AR | √ | - | *SERPINB6* | 173321 | AR | √ | √ |
| *COL9A1* | 120210 | AD | √ | - | *KCNE1* | 176261 | AD | √ | √ |
| *COL9A2* | 120260 | AD;AR | √ | - | *FGFR2* | 176943 | AD;AR | √ | - |
| *COL11A1* | 120280 | AD;AR | √ | - | *RDX* | 179410 | AR | √ | √ |
| *GJB2* | 121011 | AD;AR | √ | √ | *MYO7A* | 276903 | AD;AR | √ | √ |
| *GJA1* | 121014 | AD;AR | √ | √ | *POU3F4* | 300039 | XLR | √ | √ |
| *CRYM* | 123740 | AD | √ | √ | *SMPX* | 300226 | XLD | √ | √ |
| *DSPP* | 125485 | AD | √ | √ | *TIMM8A* | 300356 | XLR | √ | √ |
| *EDN3* | 131242 | AD;AR | √ | - | *PHEX* | 300550 | XLD | √ | - |
| *EDNRB* | 131244 | AD;AR | √ | - | *NDP* | 300658 | XLR | √ | - |
| *GATA3* | 131320 | AD | √ | - | *COL4A5* | 303630 | XLD | √ | - |
| *FGFR3* | 134934 | AD;AR | √ | - | *PRPS1* | 311850 | XLR | √ | √ |
| *FGFR1* | 136350 | AD | √ | - | *DLX5* | 600028 | AR | √ | - |
| *HGF* | 142409 | AR | √ | √ | *LRP2* | 600073 | AR | √ | - |
| *HOXA1* | 142955 | NN | √ | - | *SIX5* | 600963 | NA | √ | - |
| *IGF1* | 147440 | AR | √ | - | *MYO6* | 600970 | AD;AR | √ | √ |
| *MITF* | 156845 | AD;AR | √ | - | *SMAD4* | 600993 | AD | √ | - |
| *FOXI1* | 601093 | AR | √ | √ | *DIABLO* | 605219 | AD | √ | √ |
| *SIX1* | 601205 | AD | √ | √ | *USH1C* | 605242 | AR | √ | √ |
| *MYO1A* | 601478 | NA | √ | √ | *OPA1* | 605290 | AD | √ | - |
| *EYA1* | 601653 | AD | √ | - | *TMPRSS3* | 605511 | AR | √ | √ |
| *DIAPH1* | 602121 | AD;AR | √ | √ | *PCDH15* | 605514 | AR | √ | √ |
| *SNAI2* | 602150 | AD;AR | √ | - | *CDH23* | 605516 | AR | √ | √ |
| *ESRRB* | 602167 | AR | √ | √ | *CLDN14* | 605608 | AR | √ | √ |
| *KCNJ10* | 602208 | AR | √ | √ | *SLC26A4* | 605646 | AR | √ | √ |
| *SOX10* | 602229 | AD | √ | - | *WFS1* | 606201 | AD;AR | √ | √ |
| *POU4F3* | 602460 | AD | √ | √ | *ESPN* | 606351 | AD;AR | √ | √ |
| *TECTA* | 602574 | AD;AR | √ | √ | *CLRN1* | 606397 | AR | √ | √ |
| *TNFRSF11B* | 602643 | AR | √ | - | *BSND* | 606412 | AR | √ | √ |
| *MYO15A* | 602666 | AR | √ | √ | *STRC* | 606440 | AR | √ | √ |
| *GPR98* | 602851 | AD;AR | √ | √ | *PAX3* | 606597 | AD;AR | √ | - |
| *COCH* | 603196 | AD | √ | √ | *TMC1* | 606706 | AD;AR | √ | √ |
| *PTPRQ* | 603317 | AR | √ | √ | *MYO3A* | 606808 | AR | √ | √ |
| *GJB3* | 603324 | AD;AR | √ | √ | *ALMS1* | 606844 | AR | √ | - |
| *KCNQ4* | 603537 | AD | √ | √ | *TCOF1* | 606847 | AD | √ | - |
| *EYA4* | 603550 | AD | √ | √ | *OTOA* | 607038 | AR | √ | √ |
| *OTOF* | 603681 | AR | √ | √ | *TMIE* | 607237 | AR | √ | √ |
| *SLC19A2* | 603941 | AR | √ | - | *PDSS1* | 607429 | AR | √ | - |
| *GJB6* | 604418 | AD;AR | √ | √ | *KCNQ1* | 607542 | AD;AR | √ | √ |
| *OTOG* | 604487 | AR | √ | √ | *SLC17A8* | 607557 | AD | √ | √ |
| *HOXA2* | 604685 | AD;AR | √ | - | *USH1G* | 607696 | AR | √ | √ |
| *SLC26A5* | 604943 | AR | √ | √ | *TJP2* | 607709 | AR | √ | √ |
| *DFNB31* | 607928 | AR | √ | √ | *DIAPH3* | 614567 | AD | √ | √ |
| *SOX9* | 608160 | AD | √ | - | *CEACAM16* | 614591 | AD | √ | √ |
| *SEMA3E* | 608166 | AD | √ | - | *SERAC1* | 614725 | AR | √ | - |
| *USH2A* | 608400 | AR | √ | √ | *MT-RNR1* | 561000 | Mito | √ | √ |
| *MYH14* | 608568 | AD | √ | √ | *MT-TS1* | 590080 | Mito | √ | √ |
| *GRHL2* | 608576 | AD;AR | √ | √ | *MT-TE* | 590025 | Mito | √ | - |
| *GIPC3* | 608792 | AR | √ | √ | *MT-TK* | 590060 | Mito | √ | - |
| *DFNA5* | 608798 | AD | √ | √ | *MT-TL1* | 590050 | Mito | √ | - |
| *CHD7* | 608892 | AD | √ | - | *CCDC50* | 611051 | AD | √ | √ |
| *GPSM2* | 609245 | AR | √ | √ | *MIR96* | 611606 | AD | √ | √ |
| *LHFPL5* | 609427 | AR | √ | √ | *LRTOMT* | 612414 | AR | √ | √ |
| *ILDR1* | 609739 | AR | √ | √ | *PDZD7* | 612971 | AR | √ | √ |
| *TRIOBP* | 609761 | AR | √ | √ | *LOXHD1* | 613072 | AR | √ | √ |
| *SLC4A11* | 610206 | AR | √ | - | *GRXCR1* | 613283 | AR | √ | √ |
| *DFNB59* | 610219 | AR | √ | √ | *TPRN* | 613354 | AR | √ | √ |
| *MARVELD2* | 610572 | AR | √ | √ | *MSRB3* | 613719 | AR | √ | √ |
| *SOBP* | 613667 | AR | √ | - |  |  |  |  |  |

√ denotes that genes are included, whereas - denotes genes are excluded.

AR, autosomal recessive. AD, autosomal dominant. XLD, X-linked dominant. XLR, X-linked recessive. Mito, Mitochondrial.
