# Supplementary material for: Increased diagnostic yield by reanalysis of data from a hearing loss gene panel
Source: BMC Med Genomics. 2019 May 28;12:76. doi: 10.1186/s12920-019-0531-6 (PMC6540452; doi:10.1186/s12920-019-0531-6)
Supplement: Supplementary file 2 — Table S2. Molecular characteristics of diagnosed patients. (DOCX 26 kb) [file 12920_2019_531_MOESM2_ESM.docx]

**Table S2 Molecular characteristics of diagnosed patients**

| Characteristic | Before reanalysis, No. (%) (n=82) | After reanalysis, No. (%) (n=90) |
| --- | --- | --- |
| Gene |  |  |
| *GJB2* | 37 (45) | 37 (41) |
| *SLC26A4* | 29 (35) | 29 (32) |
| Others | 16 (20) | 24 (27) |
| Inheritance |  |  |
| AR | 71 (87) | 77 (86) |
| AD | 4 (5) | 7 (8) |
| XLD | 1 (1) | 0 (0) |
| XLR | 1 (1) | 1 (1) |
| Mito (Maternal) | 2 (2) | 2 (2) |
| Multiple | 3 (4) | 3 (3) |

AR, autosomal recessive. AD, autosomal dominant. XLD, X-linked dominant. XLR, X-linked recessive. Mito, Mitochondrial.
